# A move toward patient dissatisfaction? A critical review of the disproportionate focus on patient satisfaction compared to dissatisfaction

**DOI:** 10.1186/s41687-026-01070-9

**Published:** 2026-04-18

**Authors:** Henrik Pedersen, Jonas Vaag, Audun Havnen, Mariela L. Lara-Cabrera

**Affiliations:** 1 Norwegian University of Science and Technology (NTNU), Trondheim, Norway; 2 Inland Norway University of Applied Sciences, Lillehammer, Norway

**Keywords:** Patient satisfaction, Patient dissatisfaction, Satisfaction with treatment, Dissatisfaction with treatment, Healthcare quality, Quality assessment, Patient-centered care

## Abstract

**Background:**

Patient satisfaction has been a widely used construct when measuring the patient perspective and a vital component of both patient-centered care and quality assurance. Nevertheless, several concerns regarding patient satisfaction have been raised in the literature. These include lack of definitional consensus and measurement standardization, consistently high satisfaction scores, and ceiling effects. Another concern is that striving for high satisfaction may incentivize pleasing the patient rather than delivering the highest-quality care.

**Objective:**

This critical review clarifies the concept of patient satisfaction, primarily by distinguishing it from related concepts such as patient experience and patient-centered care. The primary goal is to explore issues surrounding patient satisfaction and investigate its relationship with the underexamined concept of patient dissatisfaction. The discussion focuses on underlying problems and potential pathways toward improved usage and understanding of the patient satisfaction construct.

**Methods:**

Given the conceptual nature of these topics, which are not easily addressed by a single research question, we chose an unsystematic critical review for its flexibility in addressing theoretical and methodological issues.

**Discussion:**

Depending on the context and objective of its measurement, investigating and minimizing patient dissatisfaction may be more appropriate and may resolve many of the concerns raised by critics of patient satisfaction. This contrasts with current practices, which primarily focus on investigating and maximizing satisfaction. Thus, a complementary focus on patient dissatisfaction may offer utility for quality improvement. Finally, the article presents unresolved conceptual issues, along with suggestions for future research.

**Conclusion:**

Patient dissatisfaction has remained a largely overlooked blind spot in patient satisfaction research and assessment. Resolving conceptual issues surrounding patient satisfaction and dissatisfaction and developing improved methods to identify pernicious dissatisfaction may render satisfaction scores more meaningful, thereby enhancing future quality-assurance evaluations.

## Introduction

Over the last couple of decades, patient satisfaction measures have emerged as one of the most common methods for assessing patient perceptions. Several factors have contributed to this widespread adoption, including its origins in consumer research, the rise of “new public management”, and the “health consumer movement”, and this movement’s connections to other patient rights movements [1, 2]. These factors have ultimately led to patient satisfaction becoming a common indicator of quality of care [3, 4], an indicator of intervention acceptability, and a concept intimately connected to patient-centered care [5]. Indeed, many countries have made patient satisfaction measurement mandatory as part of continuous quality monitoring [6, 7]. Furthermore, international organizations such as the World Health Organization and the Organization for Economic Co-operation and Development also emphasize its importance [8].

Additionally, patient satisfaction is already playing a major role in newer frameworks for healthcare improvement, such as the Quintuple aim [9], which strive to improve the patient experience (1), population health (2), cost-effectiveness (3), clinician wellbeing (4), and health equity (5). Here, the subjective nature of the patient satisfaction construct is a strength, as it may help inform processes aimed at enhancing the patient experience, improving treatment outcomes, and protecting less privileged populations from adverse treatment trajectories [3, 10, 11]. However, this is ultimately dependent on how the construct is conceptualized, measured, and put to use.

In this critical review, we begin by clarifying the concept of patient satisfaction, primarily by disambiguating it from related terms and summarizing the construct’s most common validity concerns raised by its critics. We then argue that investigating and minimizing the relatively unexplored concept of patient dissatisfaction may, in many cases, be more appropriate. This contrasts with current practices, which mainly revolve around investigating and maximizing high satisfaction.

Moreover, this paper uses an unstructured critical review format. We identified relevant literature through an iterative process that began with exploratory searches in collaboration with a research librarian across the databases Medline and Embase. These searches used “patient satisfaction” and “patient dissatisfaction” as main concepts, along with various free-text terms, to identify articles discussing the definitions of these constructs or raising validity concerns about their operationalization, use, or theoretical foundations. This was followed by more goal-oriented searches of the reference list and citations of key articles discussing theoretical issues with the patient satisfaction construct or patient dissatisfaction. These key articles were identified through discussion between the first and last author.

Rather than summarizing existing findings, this review’s objective was to clarify the concept of patient satisfaction, primarily by distinguishing it from related concepts such as patient experience and patient-centered care. Furthermore, it aimed to identify blind spots in the literature, explore and thoroughly discuss underlying problems, and suggest potential approaches to improve the use and understanding of the patient satisfaction construct. These matters are not easily addressed by, or condensed into, a concrete research question, or by a synthesis of existing findings based on strict inclusion and exclusion criteria. Therefore, we chose the unsystematic format when addressing the issues presented in this paper.

### Patient satisfaction

Patient satisfaction can be measured in several ways: through informal conversations with hospital staff, or through more structured conversations, meetings, or interviews. However, in research and in systematized quality assessment, the use of closed-ended questionnaires is the norm [12, 13]. Sometimes, with the addition of open-ended questions for patients to provide free-text comments [14, 15].

One critique of patient satisfaction is the lack of consensus on a specific definition [1, 7, 13, 16]. Studies examining patient satisfaction often do not define it at all or define it in straightforward, naïve, or tautological ways (e.g., patient satisfaction is how satisfied a patient is with the services they received) [1]. One narrow definition, however, states that patient satisfaction is achieved to the extent that “services gratify the patient’s wants, wishes, or desires for treatment” [17] (p. 212). Other definitions have largely focused on what factors lead to, or are consequences of, satisfaction and dissatisfaction. In this respect, patient satisfaction is viewed both as a valuable outcome and as a process variable [12, 13, 15, 16, 18, 19]. This stems from evidence linking satisfaction to treatment adherence and positive treatment outcomes, and dissatisfaction to premature treatment termination, making it an important goal during treatment [12, 13, 15, 16, 18, 19]. Furthermore, patient satisfaction after treatment is associated with respecting patients’ involvement in treatment decisions [20] and providing health information about disease and treatment to facilitate such participation [18, 21]. It has also been linked to wait-list duration [21], the treatment facilities themselves, and the perceived technical ability and emotional warmth of hospital staff [18].

Although a specific, agreed-upon definition is lacking in the literature, the general consensus is that patient satisfaction, broadly speaking, is a subjective evaluation of how patients experienced the treatment, which, in turn, is highly dependent on their expectations [1–3, 12, 13, 17, 22–25]. However, there is less agreement regarding the exact relationship between these two concepts [1, 2, 12, 24, 25]. Part of this disagreement concerns how exactly “expectations” should be operationalized, and whether other constructs, such as care preferences, apprehensions about the treatment process, or perceived and expressed needs, are expectations or something else entirely. Coyle and Williams [25] exemplify this by highlighting findings from Driedger [26], who found that women’s treatment satisfaction after childbirth was determined by whether the experience was worse or better than expected. This is not simply a matter of whether their expectations were met or not, but whether their apprehensions were realized. These apprehensions, in turn, are difficult to translate directly into expectations comparable to others, such as the expectation that the staff is respectable. For example, McGregor has suggested that dissatisfaction partly results from unrealistic expectations that are hard or impossible to meet [27]. These apprehensions, however, behave oppositely. Although they may be unreasonable and unrealistic, not realizing them leads to satisfaction rather than dissatisfaction.

### Disambiguation of related terms

Given the absence of a clear definition of patient satisfaction in the literature [1, 2, 7, 13, 16, 28], an alternative approach to further clarify the concept is to disambiguate it from related terms. Two concepts closely associated with patient satisfaction are patient-centered care and patient experience. A common criticism is that these terms are often used interchangeably [1, 23, 29]. Nevertheless, this should not significantly affect the argument presented here.

Patient-centered care refers to a quality of professional organizational and personal relationships in health care, that puts the patient “at the heart” of health services [30]. It is an approach in which patients’ needs are sought to be understood and respected, and in which patients are encouraged to play an active role in their treatment. Thus, the main difference between patient-centered care and patient satisfaction is that patient-centered care refers to both how health services are organized and how this relates to the patient role. In contrast, patient satisfaction refers to a particular set of a patient’s internal states. In other words, patient satisfaction refers to a subjective *response* to the services received, rather than an evaluation of specific aspects of the services themselves [29] or the values that underpin them. This distinction is perhaps clearest in situations where patients express strong preferences for treatments that are deemed unnecessary or outright harmful by the health care provider. Here, acquiescing to the patients’ demands violates professional integrity, and the healthcare provider should decline to fulfil the patients’ request [31]. Situations like this may leave patients dissatisfied, but it need not affect an overarching process that brings patient preferences to the fore and values patient participation [29].

In general, patient experience measures report what patients have encountered during their care; in other words, these measures focus on patients’ views of what actually occurred, rather than a subjective evaluation of different aspects of their care [32]. An example of a statement found in a patient experience questionnaire is “I received information about treatment options for my mental health problems” [23] (p. 2154). Conversely, a satisfaction questionnaire might include the question “Do you consider that your treatment has been adjusted to your situation?” (p.2154) [23]. Despite this distinction, distinguishing the two terms in practice is difficult, as most patient experience questionnaires include both concepts and researchers tend to use the terms interchangeably [23]. Indeed, as Fernandes et al. have pointed out, there are no MeSH terms for “patient experience” in PubMed. Many “patient experience-articles” are therefore categorized wrongly under “patient satisfaction” [23].

### Validity concerns surrounding patient satisfaction raised by critics

Alongside its popularity, several concerns have been raised in the literature about the validity of the patient satisfaction construct. For the most part, these concerns relate to properties of patient satisfaction questionnaires or to how scores generated by these questionnaires are used. This is most likely because patient satisfaction questionnaires are the most common way of operationalizing the construct, by far [12, 13]. The concerns include a lack of measure standardization; satisfaction as a sole indicator of quality; consistently high satisfaction scores and ceiling effects on patient satisfaction measures; the role of patients’ expectations and subjectivity in the score’s formation; and concerns that striving towards high satisfaction scores may be more related to pleasing the patient than delivering quality care.

Lack of standardization is a problem consistently pinpointed by systematic reviews examining different aspects of patient satisfaction [1, 10, 12, 13, 16, 33–35]. Nevertheless, this is not an issue exclusive to patient satisfaction research, and it is not due to a lack of validated patient satisfaction measures [12, 13]. This issue arises from the tendency to make ad hoc measures rather than using existing validated measures or validating existing measures in a new context. Another second-order standardization issue concerns how to interpret and present patient satisfaction scores, even when using validated measures. However, the scope of this potential issue has not yet been investigated. One example of this is presented in the section “Patient satisfaction and the acceptability of individual interventions.”

In turn, a lack of standardization can potentially creep into the next concern: high satisfaction scores and ceiling effects [10, 17, 23, 33, 36]. Without proper norms, comparisons between studies become difficult. It has long been recognized that most studies report high satisfaction scores [17, 25, 33, 36]. Isolated scores, especially generated from unvalidated measures, may therefore give an inflated sense of patient satisfaction. Regarding ceiling effects, one remedy may be to expand the range of response options for measurement items [15] or, as discussed further below, to measure patient satisfaction across multiple domains [2, 22, 33] rather than only global satisfaction. It is worth noting, however, that ceiling effects are a contextual issue [37] and are relevant only if the goal of patient satisfaction measurement is to differentiate high levels of satisfaction.

The subjective nature of the patient satisfaction construct and its relationship to patients’ expectations have led some researchers to question its validity as a sole indicator of care quality. Fernandes et al. argue that the usefulness of satisfaction scores is limited because “two patients who receive the same care but who have different expectations may not express the same degree of satisfaction.” [23] (p. 2154). Sheppard raises similar concerns, noting that satisfaction scores may be more closely related to a patient’s expectations and cultural background than to the actual treatment experience (what happened?) [38]. Another focus of criticism has been clinically unfeasible or unrealistic expectations [23, 27, 29, 31], where, according to critics, low satisfaction scores can arise despite high-quality care. Proponents of patient satisfaction do not deny these objections but instead argue that this highlights the need for its measurement: “Satisfaction should be seen as a unique evaluative perspective, necessary but not sufficient by itself in the assessment of care, which cannot be a substitute for other information such as psychopathology or functioning” [13] (p. 44). Patient satisfaction should not replace multifaceted quality assessments guided by overarching theoretical frameworks, but it nonetheless is an essential part of such assessments [3].

In other words, we have to acknowledge that the care a patient receives is always an interaction between psychological factors within the patient and aspects of the care itself. Additionally, patients who express “unreasonable expectations” may be at risk of adverse treatment trajectories and of formally complaining [18, 25, 39]. They may also risk losing trust in the institution responsible for their care [40]. Their dissatisfaction, therefore, may be viewed as a communication issue – a failure to understand their preferences and expectations [3, 27], and subsequently to moderate these to represent clinical reality [3, 10].

Critics, in turn, argue that if the goal is to maximize satisfaction scores through expectancy moderation and the meeting of patients’ needs and preferences, this risks pleasing patients to the extent that quality suffers [29, 31]. However, the view mentioned above, where patient “Satisfaction should be seen as a unique evaluative perspective; necessary but not sufficient by itself in the assessment of care, which cannot be a substitute for other information such as psychopathology or functioning” [13] (p. 44) may inoculate against this tendency. On the other hand, some have concluded the opposite, that higher satisfaction is associated with more adequately used health resources [18]. Currently, this issue is difficult to evaluate fully, and further research is necessary. Most of these limitations and validity concerns, however, may be a product of a disproportionate focus on patient satisfaction compared to patient dissatisfaction.

### Patient dissatisfaction

Despite the popularity of patient satisfaction, patient dissatisfaction has received surprisingly little attention [25]. As of 28th February 2026, searching through titles and abstracts in PubMed mentioning “patient satisfaction” or “patient dissatisfaction” reveals a forty-fold difference: 60.527 records include “patient satisfaction” and only 1.491 records include “patient dissatisfaction”.

Proponents of focusing on patient dissatisfaction argue that patient satisfaction assessment is important, but that shifting the focus to dissatisfaction will address many of the validity problems mentioned above. Of particular interest is the ambiguity surrounding high satisfaction scores [22, 37, 38], which has led some researchers to suggest that satisfaction and dissatisfaction are not opposite poles of a single continuum, but rather two distinct constructs [25, 41]. Such a discovery would be very impactful to patient satisfaction research, as patient dissatisfaction is currently de facto conceptualized as low scores on patient satisfaction measures.

The main reason for this two-factor hypothesis is that high satisfaction does not necessarily imply the absence of dissatisfaction. Patients may still be dissatisfied with specific aspects of care, even though they express high overall satisfaction [25]. This hypothesis suggests that the drivers of dissatisfaction (e.g., poor communication and long waiting times) may differ from those of high satisfaction (e.g., feeling involved and respected). Making patient satisfaction and patient dissatisfaction analogous to Herzberg’s theory of motivation [42], which makes a distinction between drivers that generate motivation, and the drivers that alleviate dissatisfaction, of which Herzberg calls hygienic factors. If patient dissatisfaction and patient satisfaction follow the same conceptual structure – and dissatisfaction and satisfaction have different, separate determinants – this has clear implications for how they should be operationalized and put to use. To date, whether patient satisfaction and dissatisfaction are best conceptualized as two poles of a single construct or as distinct constructs remains an unanswered question.

Another way of conceptualizing the ambiguity of high patient satisfaction scores is as a tension between domain-specific patient satisfaction and dissatisfaction, and general patient satisfaction and dissatisfaction. In this view, patient satisfaction and dissatisfaction are opposite poles of the same construct, but a person has several domain-specific satisfaction levels. This idea is analogous to Bandura’s well-known discussion of self-efficacy, which he did not consider a general trait but rather a concept linked to distinct realms of functioning [43]. Bandura argued that a person could have high self-efficacy in one domain, such as a profession, while having low efficacy in another, like social settings. Similarly, patient satisfaction and dissatisfaction may be domain-specific; patients may be satisfied with the technical aspects of their treatment but dissatisfied with interpersonal communication [3]. Indeed, when asked, many “highly satisfied patients” also provide suggestions for improvement. Lee et al. interviewed 976 patients after discharge, of which 45% offered suggestions for improvement [44]. Even the simple addition of open-ended questions to satisfaction questionnaires in routine care, prompting suggestions for improvement, can lead to a third of patients providing suggestions in free text [15], despite reporting high levels of global satisfaction on the closed-ended items Moreover, when Rudman et al. [45] examined both domain-specific and global evaluations with intrapartum care, they found that analyzing patient satisfaction on a domain-specific level revealed diverse patterns of satisfaction and dissatisfaction for most patients. Thus, looking at patients’ satisfaction levels with specific domains of care presented a more nuanced picture of patients’ views that the global patient satisfaction measure seemed to mask [45]. This finding contrasts with the commonly reported pattern that as many as 70–90% of patients report high overall satisfaction [25].

Moreover, patients’ explanations for their dissatisfaction seem less ambiguous [25, 46], more concrete and detailed, and have a clearer source than explanations of high patient satisfaction [41]. Findings such as these have led researchers to quip, “Satisfied patients are all alike; every dissatisfied patient is dissatisfied in their own way.” [47] (p. 3202), paraphrasing Tolstoy’s Anna Karenina. However, it does seem that dissatisfied patients are all alike in one particularly important respect: their dissatisfaction is a clear indication that *something* has gone wrong in the interaction between them and the health services. In contrast, high satisfaction does not necessarily indicate that everything went well. The relative importance of patient satisfaction and dissatisfaction scores, however, ultimately depends on the reasons and goals behind their measurement, regardless of whether it is measured for scientific, clinical, or quality assessment purposes.

### What are the goals of measuring satisfaction?

The utility of satisfaction and dissatisfaction scores ultimately depends on the goals behind their measurement and the context in which they are measured. This section outlines and discusses common reasons for measuring patient satisfaction. First, we explore differences in goals between privatized and governmental healthcare. These contrasting priorities may lead to differences in how patient satisfaction should be measured and evaluated in these contexts, particularly if the goal is to use it as part of continuous quality monitoring. Second, we explore how patient satisfaction is often used as a proxy for acceptability when evaluating individual psychiatric or other medical interventions. We then discuss how most current research practices report satisfaction scores in this context, highlight their limitations, and propose improving them by shifting the focus to dissatisfied patients instead. Third, we discuss how high patient satisfaction scores may be ambiguous by themselves, compared to dissatisfaction, when evaluating changes in clinical practice. This is illustrated through hypothetical scenarios in which patient satisfaction may decrease despite improvements in “objective” quality.

### Differences between governmental and privatized healthcare

There may be subtle differences in the objectives behind patient satisfaction assessment in privatized and public or governmental health services that are worth mentioning here. Although all healthcare clinics strive to achieve excellent health outcomes, a private clinic’s additional goal is “consumer retention”: getting consumers to return when they need similar services [1, 18, 48]. Governmental or public healthcare services, on the other hand, should theoretically be more concerned with whether the patient will seek any relevant healthcare [49].

This leads to some differences in practice. Private clinics naturally aim to achieve high patient satisfaction scores relative to other clinics, thereby increasing consumer loyalty, recommendations of their services to others, and “behavioral intention” – the intention to use their services in similar scenarios in the future. Indeed, this has been the aim of several service quality models, satisfaction models, and related patient satisfaction research [1, 18, 50], which may be due to the concept’s origin in consumer research [1, 2]. In a highly competitive privatized marketplace, achieving high satisfaction scores is crucial, ideally higher than competitors’ [48]. Consequently, measures without ceiling effects that can differentiate between higher levels of satisfaction are necessary.

Governmental organizations or public clinics, on the other hand, may not be as concerned with where a patient seeks help and should therefore prioritize satisfaction scores high enough to avoid negative consequences. Such consequences include dropping out of treatment, not adhering to prescribed treatment or agreed-upon interventions, not seeking adequate treatment, fewer malpractice litigations, less frequent provider change leading to inefficiencies, or losing trust in the healthcare system [18, 25, 39, 40]. This may also include not seeking treatment at all [49], as well as indirect consequences such as failing to motivate a reluctant family member to seek treatment despite their apprehensions. Furthermore, reducing these consequences is especially important in vulnerable populations who do not have the opportunity to choose between clinics or hospitals [10]. In such cases, the difference between high and excellent satisfaction levels becomes less important, and it becomes more important to focus on identifying dissatisfaction and addressing its causes. Ceiling effects, then, become much less of a concern in this context.

### Patient satisfaction and the acceptability of individual interventions

Patient satisfaction is also frequently measured as a proxy for the acceptability of specific treatment interventions – defined as “judgments by lay persons, clients, and others of whether treatment procedures are appropriate, fair, and reasonable for the problem or client” [51] (p. 483). Here, aggregated scores, often highlighting high satisfaction, are the norm. Two illustrative quotes from published randomized controlled trials include, “*The mean score was 28.8 [out of 32] (SD 2.4; range 25–32)*,* implying a high satisfaction with the CBT [cognitive behavioural therapy] intervention*.” [52] (p. 991), and “*Treatment group participants who completed the post-treatment satisfaction questionnaires reported a moderate level of satisfaction with the overall program*,* with 27/38 (71%) reporting being either very satisfied or mostly satisfied*,* and 11/38 (29%) neutral/somewhat satisfied*,* with no participants rating the program as unsatisfactory*.” [53] (p. 896). First, although both studies use the same validated satisfaction questionnaire, comparability is compromised by a lack of reporting standards. Second, the high scores, which are the norm rather than the exception, also stand out. Is it possible to discern which intervention has higher acceptability? We argue that if the goal is to evaluate acceptability, distinguishing between high and excellent scores should be of lesser importance. Indeed, two studies reporting identical scores may have vastly different acceptability.

Consider, for example, the latter study mentioned above: an evaluation of a transdiagnostic internet intervention for anxiety disorders [53]. Suppose the 29% of patients who reported being “neutral/somewhat satisfied” were asked to further elaborate on their responses. If the most common reason behind their responses was “the intervention was not that relevant to my problems”, this would lead to a vastly different conclusion than if their reason was “I had problems logging into the digital platform on several occasions”. Here, exploring the reasons for “dissatisfaction” yields much more information, both about the current intervention and potential further development. Of course, one could interview everyone, though this would take considerably more time. Moreover, as stated above, the answers from satisfied participants may provide little informational value due to their vagueness and lack of detail [41].

### The ambiguity of satisfaction scores when evaluating changes in practice

To further illustrate another way in which patient satisfaction can be ambiguous, scenarios can be hypothesized in which satisfaction decreases while quality “objectively” increases. For example, a clinic that has been overprescribing benzodiazepines or painkillers for some time, but has recently reduced prescriptions to align with recommended guidelines, may experience a decline in patient satisfaction scores [54]. Secondly, triage practices may result in some patients being discharged earlier than expected during unexpected increases in patient influx, potentially leading to lower satisfaction scores. Indeed, “too early” discharge is a commonly expressed reason for patient dissatisfaction [39]. Third, a clinic may experience a drop in patient satisfaction after increasingly starting to suggest unwanted lifestyle changes, such as weight loss, or a reduction in drinking and/or smoking.

The main theme across these hypothetical situations is that some practice changes that are good for the patient population may be perceived negatively by particular individuals or patient groups. Others include changes that are good in the long term but may conflict with short-term patient preferences. All examples underline the importance of evaluating the reasons behind a patient’s satisfaction and/or dissatisfaction to get a complete picture of what the scores mean [3]. However, most patient satisfaction measures do not clearly indicate the reasons behind expressed satisfaction or dissatisfaction [13]. A further complementary investigation may therefore be necessary to get a complete picture when thoroughly evaluating changes in practice [3]. In this context, investigations of patients identified as dissatisfied, or “dissatisfaction scores,” rather than “satisfaction scores,” may have some advantages.

First, they are most likely fewer in number. Second, the source of dissatisfaction may be more concretely expressed than the reasons for satisfaction [41]. A targeted, complementary investigation of this group may therefore take less time, while also being more informative. Third, as discussed above, each expression of dissatisfaction indicates that *something* has gone wrong in the interaction between the hospital and the patient. Satisfaction, however, does not symmetrically indicate that everything has gone well. Fewer dissatisfied patients may therefore be a stronger indicator of high-quality care, rather than a higher aggregated mean on patient satisfaction measures. Moreover, further exploration of expressed dissatisfaction may reveal that the dissatisfaction is directly related to the changes in practice that have presumably enhanced the quality of care. For example, more appropriate prescribing of benzodiazepines or painkillers. In such cases, what went wrong may be viewed as poor expectation modulation, communication, or insufficient information provided to patients in relation to the change [3], all of which are major determinants of patient satisfaction and dissatisfaction [12, 18, 25, 55]. Indeed, having an assigned healthcare provider and receiving proper patient education appear to be beneficial for patient satisfaction when reducing painkiller use [56, 57]. Still, more research is needed on these types of precarious situations to fully understand what characterizes them, identify potential vulnerable patient groups, and determine how to mitigate their potential adverse effects.

Lastly, reasons for dissatisfaction theoretically have the potential to be more serious and have more severe consequences than high satisfaction. (e.g., loss of trust in the healthcare system, reduction in future help-seeking behavior). This asymmetry is important because one can easily imagine a scenario in which mean satisfaction scores increase, but the proportion of dissatisfied patients also rises, due to polarization in the score distribution. In such a scenario, the total number of adverse treatment trajectories may increase while aggregated mean satisfaction scores also improve. This also relates to the point made above about privatized and governmental healthcare, as this may, in some cases, be beneficial for private clinics, provided satisfied patients remain loyal and dissatisfied patients do not pursue litigation or publish widely seen negative feedback.

### Implications and future research

The aim of measuring patient satisfaction has often been to maximize satisfaction scores, leading critics to highlight potential ceiling effects in these measures and the ambiguity of high satisfaction scores. Although maximizing satisfaction may be an important goal in some cases, we have argued that if the objective is to assess the acceptability of an intervention, evaluate changes in practice, or reduce adverse treatment trajectories, then exploring and minimizing dissatisfaction may be more appropriate.

From a clinical and research perspective, several strategies may help identify patient dissatisfaction, according to current research. First, the use of domain-specific measures may help clinicians and health organizations identify nuances in patients’ subjective evaluations of care that global measures seem to mask [45]. Second, it may be beneficial to explicitly ask about grievances or suggestions for improvement through open-ended questions added to satisfaction questionnaires or in interviews [3, 35, 40, 41], as high satisfaction does not seem to indicate that patients lack ideas for improvement [15, 44]. Even though analyzing qualitative data is more resource-intensive, it may yield important insights into patients’ evaluations that closed-ended questionnaires might miss. However, the depth of qualitative data collection (e.g., interviews versus open-ended written questions added to questionnaires) and analysis will depend on context-specific factors related to costs versus potential benefits. Nevertheless, further analysis should prioritize patients identified as dissatisfied on existing patient satisfaction measures, as current research suggests that this group is more likely to have experienced suboptimal care and is at risk of adverse treatment trajectories. Further research into innovative approaches, such as sentiment analysis conducted by artificial intelligence [55], may also have great potential in this context.

At the same time, it is also worth stressing that even proponents of patient satisfaction agree that it is not a substitute for a comprehensive assessment of quality of care, using overarching multifaceted theoretical frameworks [23]. Nevertheless, its unique perspective on how patients subjectively experience their treatment is important because it influences both current and future interactions with health care services [3].

To fully understand the phenomenon of patient satisfaction and dissatisfaction, however, future research should address some conceptual issues. First, the field should acknowledge that patient dissatisfaction is a poorly defined and understudied concept. Second, future research should further explore patients’ expectations of treatment and their relationship with patient satisfaction and dissatisfaction. Due to the apparently nebulous relationship between expectations and (dis)satisfaction, another more encompassing conceptualization may be necessary, one that tries to conceptualize a patient’s broader *understanding* of the treatment context, rather than focusing solely on their expectations. Here, Donabedian’s concept of “perspective divergence” may be more appropriate [3].

Third, it is important to ask when dissatisfaction becomes a problem in treatment. We propose that it becomes a problem when it is *pernicious*: in other words, when it has negative consequences, such as loss of trust in the healthcare system or a lack of future help-seeking behavior or intentions. However, pernicious dissatisfaction remains largely unexplored outside the context of minimizing formal complaints and avoiding litigation (see Mostafapour et al. [55] for an example). This also represents a change in how patient satisfaction and dissatisfaction should be quantitatively measured and reported in certain contexts. Instead of reporting aggregated satisfaction scores, future research should establish cut-offs for pernicious patient dissatisfaction and report the frequency of dissatisfied patients. Questions about pernicious patient dissatisfaction are also tied to whether dissatisfaction is a separate construct from satisfaction, as discussed elsewhere in this review. If this is the case, there may be independent factors specific to dissatisfaction that are not directly related to satisfaction. However, this needs further examination.

Dissatisfaction also requires further examination at both group and individual levels. Questions remain, such as what factors in treatment processes lead to more dissatisfied patients, and whether there are patient variables that place certain individuals at risk of becoming dissatisfied with their care across healthcare contexts. Addressing such questions may help identify vulnerable populations at risk of the adverse treatment trajectories discussed throughout the review.

Moreover, the sensitivity of existing patient satisfaction measures in identifying meaningful levels of dissatisfaction must be further explored. Conceptually, most of these measures are designed to quantify the degree to which a patient is satisfied with their treatment and may therefore risk overlooking important aspects of dissatisfaction. Research should investigate whether dissatisfaction is simply low scores on a satisfaction measure or whether it is a multifaceted construct that must be understood separately from patient satisfaction and in terms of both internal factors and the external context of each individual patient.

The observations mentioned above, in which some researchers have noted that satisfaction and dissatisfaction may coexist, could be attributed to insensitive measures that capture only extreme negative events [58]. Another reason may be that studies do not clearly differentiate between domain-specific and general patient satisfaction, as some researchers have observed [2, 42]. Satisfaction scores tend to be lower and more variable when evaluating multiple components of care simultaneously. Whether patient dissatisfaction is the opposite of patient satisfaction or best conceptualized as a different construct altogether will indicate how patient dissatisfaction and patient satisfaction should be operationalized, which existing measures are appropriate for their measurement, and whether entirely new measures are necessary. As we have argued, however, shifting the theoretical focus to patient dissatisfaction may be beneficial for exploring and answering these questions.

Finally, concerns exist in the literature about patients not sharing their grievances about the care they have received or not filing formal complaints despite experiencing adverse events (see Gurung et al. [56], Wessel et al. [57], or Doron et al. [58] for examples). There are some methodological and ethical difficulties in this line of research, however. For example, to properly estimate the extent of underreporting, researchers must first identify patients who have reasons for complaining and then determine how many in this group filed a formal complaint or provided feedback. This means that this line of research depends on patients’ reports of their grievances from the very beginning. If some patients fear repercussions, it may be impossible to identify which patients have grounds to complain in the first place; this group may also be reluctant to participate in research. Thus, future research into patient satisfaction and dissatisfaction should not only strive to develop sensitive measures but also investigate treatment processes that prompt patients to properly evaluate their care and create opportunities to safely communicate their grievances during or after their care.

### Limitations

This review has several limitations that should be acknowledged. Our review is unsystematic and relies on informal search strategies rather than a predefined search protocol. However, we did purposely exclude non-English papers. As previously described, this approach was chosen to allow for flexibility and conceptual depth. Nevertheless, this introduces a risk of selection bias, as relevant studies or conceptual issues may have been overlooked. Our approach contrasts with scoping reviews, which also have an exploratory nature. However, scoping reviews map the field in a more systematic way and try to answer specific research questions [59].

While the existence of patient dissatisfaction is theoretically well-grounded, it remains empirically underdeveloped. As noted throughout this review, dissatisfaction has received surprisingly little attention, a gap illustrated by the difference in PubMed search results mentioned above. Consequently, several of the proposals suggested here remain speculative until further empirical research is available.

Thus, we do not view this review as definitive, and more systematic reviews of the literature, examining specific research questions related to what we have outlined here, might also be necessary. For example, there might exist subtle but important differences in psychiatric and somatic medicine - or their subfields. Our intention with this review, however, has been to foster a shift in perspective, and stimulate theoretical musings and further research in a way that does justice to the nuances of patients’ subjective evaluations of care.

## Conclusions

Despite widespread recognition of patients’ subjective assessments of treatment through patient satisfaction measures, patient satisfaction has received disproportionately more attention than patient dissatisfaction. Additionally, many researchers have raised concerns about the validity of high satisfaction scores. This critical review has argued that, depending on the goal behind its measurement, greater focus on patient dissatisfaction may address many of these validity concerns. Future researchers and quality assessors should reflect on their objective for measuring patient satisfaction and consider whether focusing on patient dissatisfaction is more appropriate. Some researchers have expressed a certain fatalism towards patient satisfaction and dissatisfaction, suggesting that dissatisfied patients will always exist [21]. However, this perspective completely overlooks the necessity to explore the reasons behind dissatisfaction to mitigate it and its potential negative consequences. Dissatisfaction indicates that something has gone wrong in the interaction between healthcare services and the patient, and the reasons behind dissatisfaction are often concrete and easily articulated. In many contexts, this makes exploring and minimizing dissatisfaction a more logical focus than striving for high satisfaction scores. Resolving conceptual issues regarding patient satisfaction and dissatisfaction and developing better ways to identify pernicious dissatisfaction may render satisfaction scores more meaningful, leading to better future quality-assurance evaluations. Until now, however, such a focus has largely remained overlooked in patient satisfaction research and assessment.

## Data Availability

No datasets were generated or analysed during the current study.
